# Sudden Bilateral Sensorineural Hearing Loss Secondary to Cerebral Venous Thrombosis

**Published:** 2018-03

**Authors:** Muhammad-Nu’aim Ishak, Nik-Mohd-Syukra Nik-Abdul-Ghani, Irfan Mohamad

**Affiliations:** 1 *Department of Otorhinolaryngology - Head and Neck Surgery, School of Medical Sciences, Universiti Sains Malaysia, Kelantan, Malaysia and Universiti Sultan Zainal Abidin, Terengganu, Malaysia.*; 2 *Department of Otorhinolaryngology - Head and Neck Surgery, School of Medical Sciences, Universiti Sains Malaysia, Kelantan, Malaysia.*

**Keywords:** Audiometry, Cerebral venous thrombosis, Sudden sensorineural hearing loss

## Abstract

**Introduction:**

Sudden sensorineural hearing loss (SSNHL) is an important otological emergency. Up to 90% of the cases are idiopathic. Cerebral venous thrombosis (CVT) is an extremely rare identifiable cause as it only represents 0.5% of all strokes.

**Case Report:**

In this paper, an unusual case of bilateral SSNHL secondary to bilateral CVT with rapid and complete recovery is reported. The patient presented with sudden bilateral hearing loss associated with some neurological symptoms. Initial computed tomography (CT) venography revealed a CVT of bilateral transverse sinuses. The patient was started on an anticoagulant and imaging was repeated after five days, revealing the absence of the thrombosis. Serial pure tone audiometry (PTA) showed complete recovery of bilateral hearing within 10 days.

**Conclusion:**

Early detection and intervention may fasten hearing recovery and improve the quality of life. The immediate restoration of venous blood flow and intracranial pressure may lead to the complete recovery of bilateral hearing loss.

## Introduction

Bilateral sudden sensorineural hearing loss caused by bilateral cerebral venous thrombosis is uncommon. The majority of sudden onset sensorineural hearing loss presents itself unilaterally, while the incidence of bilateral involvement is less than 5% (1). Sudden sensorineural hearing loss is a rare but possible symptom of cerebral venous thrombosis. It is an otological emergency which need prompt recognition and management. Early detection and intervention may fasten hearing recovery and improve quality of life.

## Case Report

A 27-year-old man came with a complaint of sudden onset bilateral hearing loss while he was attending a religious event in a mosque the night before. He was confused and unable to find his way back to his car. There was associated slurred speech, dizziness, and vomiting. Otherwise there was no ear pain, tinnitus, vertigo or ear discharge. He also denied any fever, headache or body weakness. There was no previous history of ear infection, trauma or exposure to a loud voice. He also denied taking any ototoxic medications. He had a childhood history of epilepsy with the last fitting episode occurring when he was 12-years-old. 

During his physical examination, the patient was alert and conscious. 

The vital signs were normal. Free field voice test showed that the patient had bilateral profound hearing loss. He was unable to hear a shout at 2 feet and he spoke in a loud voice with some degree of dysarthria. Tuning fork test was not administered since the patient had profound hearing loss. Otoscopic examination was also normal. He had a normal gait, no facial asymmetry, and his muscle power and reflexes were normal. 

**Fig 1:**
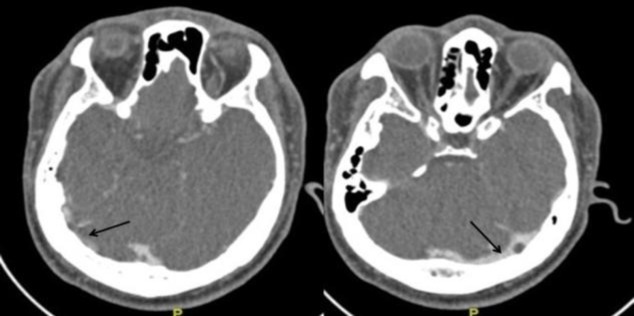
CT venography of brain (day 2 of admission) showing CVT of bilateral transverse sinuses with multifocal infarcts of various ages. Notice the dense transverse sinus due to thrombosis (arrow).

His routine blood examinations showed a haemoglobin of 135 g/L, total white cell count of 8.51 x109/L, and an ESR 3 mm/hr. His coagulation profile was normal. Screening tests for Hepatitis and Syphilis infection were negative.Based on the patient’s history and physical examination, we referred him to the neuromedical team with the diagnosis of bilateral sudden sensorineural hearing loss secondary to central causes which could be stroke or meningitis. Computed tomography (CT) followed by CT venography, which were administered on the following day, revealed cerebral venous thrombosis (CVT) of bilateral transverse sinuses and torcular herophili with multifocal infarction of various ages (Fig.1). The neuromedical team prescribed him a subcutaneous anticoagulant (fondaparinux 7.5mg daily), intravenous dexamethasone 4mg every 8 hours, and intravenous Ceftriaxone 2g daily. 

On the following day, the patient’s bilateral hearing started to improve. Free field voice test showed that he was able to hear a shout at 2 feet (80-90 dB hearing loss).

**Fig 2:**
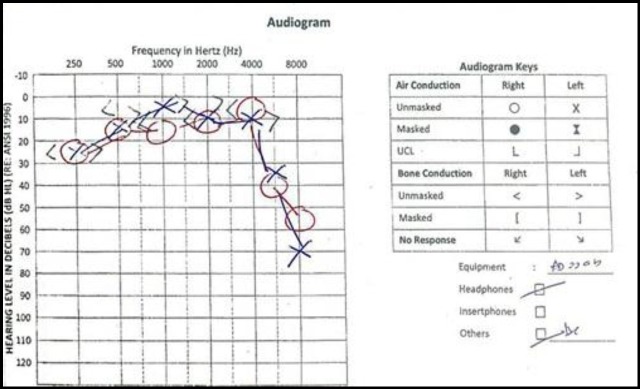
Initial audiogram on the third day of admission.

 Pure Tone Audiometry (PTA) was not performed upon admission because the patient had giddiness and was not fit for the procedure (the patient was alert but had some giddiness and instability, so the audiology staff didn’t proceed with the procedure as they afraid he might fall in PTA room). The first PTA was administered on the third day after admission, which revealed moderate-to-severe sensorineural hearing loss (SNHL) at high frequencies (4 kHz, 8 kHz) (Fig.2). 

The patient’s hearing continues to improve and he claims to have his normal bilateral hearing back 10 days after admission.

**Fig 3:**
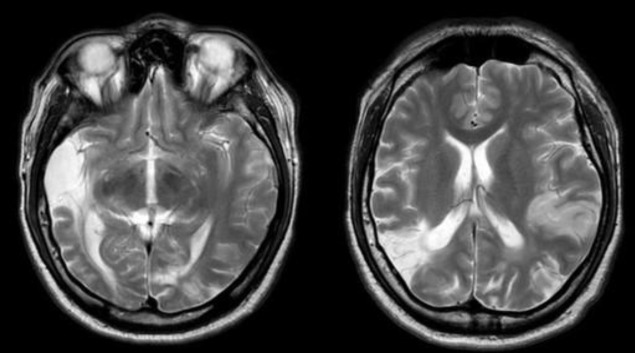
MRI brain performed after 5 days of CT scan venography, showing multifocal infarction of varying ages with no evidence of venous sinus thrombosis

Five days after the first imaging, an MRI of the brain was ordered for the patient to evaluate the progress of the CVT. The radiologist reported it as multifocal infarction of varying ages with no evidence of venous sinus thrombosis (Fig.3). 

**Fig 4:**
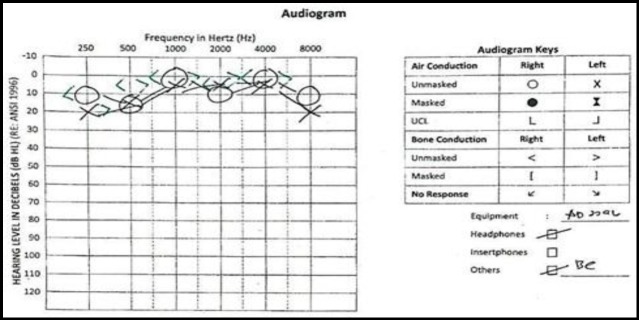
Audiogram after 10 days of admission shows full hearing recovery of bilateral ear.

The patient was discharged after 11 days with an oral anticoagulant (aspirin 75mg daily) and to complete oral prednisolone in total of 2 weeks. (the patient was discharged with oral prednisolone tapering dose - every day for 2 weeks). Four weeks post-discharge, during the patient’s ORL clinic follow-up, he claimed having normal bilateral hearing and a subsequent PTA assessment showed normal findings (Fig.4).

## Discussion

Sudden hearing loss (SHL) can be described as a rapid onset hearing impairment in one or both ears over a 72-hour period. Sudden sensorineural hearing loss (SSNHL) is a component of SHL that is sensorineural in nature and conforms to certain audiometric criteria (2). Sudden sensorineural hearing loss (SSNHL) is an otological emergency requiring prompt and thorough assessment in order to detect a possible etiology that may be life-threatening and that possesses reversible conditions. However, most of SSNHL, 85% to 90% of cases, is idiopathic (3). Idiopathic sudden sensorineural hearing loss (ISSNHL) is described as SSNHL with no established cause despite sufficient investigation.

The unilateral condition is more common and usually idiopathic. About 65% of patients which presented with a unilateral condition spontaneously recovered their hearing (4). On the other hand, the bilateral condition is rare and is usually related to serious conditions such as vascular, metabolic, autoimmune, infectious, neoplastic, traumatic, or inflammatory (5).

Cerebral venous thrombosis (CVT) has wide spectrum of clinical presentations. Classical symptoms of CVT include headache, seizures, and various focal neurological deficits including cranial nerve palsies (6). In this case, the patient presented with only sudden onset bilateral hearing loss with some degree of dysarthria without the other usual clinical presentations.

Risk factors for central venous thrombosis (CVT) include changes in hormonal levels (pregnancy, puerperium, oral contraceptives, hormone replacement therapy), mechanical causes (head trauma, catheterization, surgery, lumbar puncture), infections, cancer, acquired prothrombotic states (hyperhomocysteinemia, nephrotic syndrome), inflammatory diseases, haematological disorders, arteriovenous malformations, and drugs (7). However, in 30% of cases no underlying etiology can be identified (8). In this patient, the etiology for central venous thrombosis cannot be identified as there was no evidence (history and blood investigation) of trauma, infection or inflammatory disease.

CVT may have led to sensorineural hearing loss by inducing increased intracranial pressure. Increased pressure in the subarachnoid space may be conveyed to the perilymph and endolymphatic system via the cochlear aqueduct, causing increased fluid pressure in the internal ear which eventually causes cochlear dysfunction. Cranial nerve VIII function may also be disturbed by the increased intracranial pressure (9). Thrombosis of the transverse sinus can increase cochlear pressure and give rise to anoxic changes as a result of impaired drainage or thrombosis extending to the cochlear or labyrinthine veins (10).

In this case, CT scan with contrast and CT venography which was performed one day after admission revealed that the patient had a cerebral venous thrombosis (CVT) of the bilateral transverse sinuses and torcular herophili with multifocal infarct of various age. He was immediately started on subcutaneous fondaparinux by the neurosurgical team. We also started him on intravenous prednisolone. Within one day, his bilateral hearing started to improve evidenced by clinical hearing assessment and later by serial PTA. However, the MRI of the brain administered five days after the first CT scan revealed only multifocal infarct with absence of CVT. The probability of hearing recovery has been reported to differ according the gravity of the hearing loss threshold level upon presentation. Patients with mild hearing loss usually achieve full recovery, those with moderate hearing loss occasionally have a full recovery unless treated, and patients with severe to profound hearing loss rarely show spontaneous improvement or make a full recovery (11-13). 

However, in this case, the patient presented with profound hearing loss and continues to have improvement of bilateral hearing and even achieved a full recovery after ten days. (Fig. 4).

## Conclusion

Bilateral sudden sensorineural hearing loss is a rare presentation of cerebral venous thrombosis that is potentially reversible with prompt diagnosis and appropriate intervention. Early and accurate detection of CVT is possible with the help of CT scan and MRI. Successful management requires anticipation of related etiology, early recognition, and an optimal treatment of CVT.
